# Interaction of Avelox with Bovine Serum Albumin and Effect of the Coexistent Drugs on the Reaction

**DOI:** 10.1155/2012/408057

**Published:** 2012-02-20

**Authors:** Baosheng Liu, Chao Yang, Xiaona Yan, Jing Wang, Yunkai Lv

**Affiliations:** Key Laboratory of Medical Chemistry and Molecular Diagnosis, College of Chemistry & Environmental Science, Hebei University, Ministry of Education, Baoding 071002, China

## Abstract

The interaction between Avelox and bovine serum albumin (BSA) was investigated at different temperatures by fluorescence spectroscopy. Results showed that Avelox could quench the intrinsic fluorescence of BSA strongly, and the quenching mechanism was a static quenching process with Förester spectroscopy energy transfer. The electrostatic force played an important role on the conjugation reaction between BSA and Avelox. The order of magnitude of binding constants (*K*_*a*_) was 10^4^, and the number of binding site (*n*) in the binary system was approximately equal to 1. The binding distance (*r*) was less than 3 nm and the primary binding site for Avelox was located in subdomain IIA of BSA. Synchronous fluorescence spectra clearly revealed that the microenvironment of amino acid residues and the conformation of BSA were changed during the binding reaction. In addition, the effect of some antibiotics on the binding constant of Avelox with BSA was also studied.

## 1. Introduction

Most drugs are able to bind to plasma protein when they entrance in blood plasma system of organism, and serum albumin is the most abundant protein in blood plasma and serves as a depot protein and transport protein for numerous endogenous and exogenous compounds [1]. Generally speaking, drugs could bind with serum albumin mostly through the formation of noncovalent complexes reversibly. The drug-protein complex can be regarded as a form of drug in the biology temporary storage, it can effectively avoid drug elimination from metabolism so quickly that it can maintain the total concentration and effective concentrations of blood medicine in plasma. In addition, binding of drugs to plasma proteins controls their free, active concentrations and provides a reservoir for a longer action, the binding of drugs is responsible for the protective role of albumin. Therefore, interaction of a drug with, and competition for, the binding sites on plasma proteins might strongly affect its distribution, elimination, as well as its pharmacodynamics and toxic properties [2]. Competition between two drugs for their binding to plasma protein can strongly affect the drug disposition of both drugs, with possible serious physiological consequences. Binding parameters are indeed fundamental factors in determining the overall pharmacological activity of a drug, and in this context the determination of the binding parameters of drugs to albumin has become essential to understand their pharmacokinetic, pharmacodynamic, and toxicological profile.

**Figure 1:**
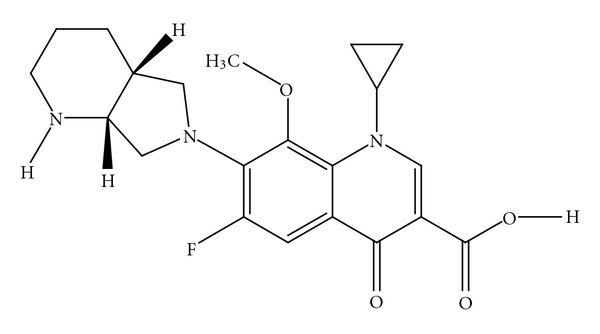
Chemical structure of Avelox.

Avelox is a new generation of fluoroquinolone antibacterial agents, which is used to treat a wide variety of bacterial infections including pneumonia, sinusitis, and worsening of chronic bronchitis (the structure is shown in Figure 1). This medicine gradually played a significant role in clinical cure. At present, the molecular interactions between BSA and many drugs have been investigated successfully in biomedical domain [3]. However, the interaction between Avelox and BSA has not been investigated, especially the effect of the common antibiotics on the binding of drugs to BSA. In this paper, we provided investigations on the interaction of Avelox with BSA by fluorescence spectroscopy under physiological pH 7.40 as well as the effect of the coexistent drugs. This study is expected to provide important insight into the essence, potential toxicity between drugs and protein in real terms, and can also provide a useful clinical reference for future combination therapy.

## 2. Experimental

### 2.1. Apparatus

All fluorescence spectra were recorded with a Shimadzu RF-540 spectrofluorophotometer and a Hitachi F-4500 spectrofluorophotometer. Absorption was measured with an UV-vis recording spectrophotometer (UV-265 Shimadzu, Japan). All pH measurements were made with a pHS-3C precision acidity meter (Leici, Shanghai). All temperatures were controlled by a CS501 superheated water bath (Nantong Science Instrument Factory).

### 2.2. Materials

Avelox (no less than 99.9% pure) was obtained from monitor of Chinese veterinary medicine. Bovine serum albumin (BSA, no less than 99% pure) was purchased from Sigma Company. Warfarin (WF), Chlorpheniramine (CP), and digitoxin (DG) were all obtained from Chinese Institute of Drug and Biological Products. Stock solutions of BSA (1.0 × 10^−4^ M) and Avelox (2.0 × 10^−3^ M) were prepared. And all the stock solutions were further diluted as working solutions prior to use. All the common antibiotics were diluted to 1.0 × 10^−3^ M. *Tris*-HCl buffer solution containing NaCl (0.15 M) was used to keep the pH of the solution at 7.40. NaCl solution was used to maintain the ionic strength of the solution. All other reagents were of analytical grade, and all aqueous solutions were prepared with newly double-distilled water and stored at 277 K.

The fluorescence intensities were corrected for the absorption of excitation light and reabsorption of emitted light to decrease the inner filter using the following relationship [4]:

(1) $$F_{\text{cor}}=F_{\text{obs}}\times e^{(A_{\text{ex}}+A_{\text{em}})/2},$$

where *F*_cor_ and *F*_obs_ are the corrected and observed fluorescence intensities, respectively. *A*_ex_ and *A*_em_ are the absorbance values of Avelox at excitation and emission wavelengths, respectively. The fluorescence intensity used in this paper was corrected.

### 2.3. Procedures

#### 2.3.1. Fluorescence Experiments

In a typical fluorescence measurement, 1.0 mL of pH = 7.40 *Tris*-HCl, 1.0 mL of 1.0 × 10^−5^ M BSA solution, and different concentrations of Avelox were added into a 10 mL colorimetric tube, successively. The samples were diluted to scaled volume with water, mixed thoroughly by shaking, and kept static for 15 min at different temperatures (293, 303, and 313 K). The excitation wavelength for BSA was 280 nm (or 295 nm), with the excitation and emission slit widths set at 5 nm. The solution was subsequently scanned on the fluorophotometer and determined the fluorescent intensity at 343 nm. The fluorescence intensity of BSA without Avelox was used as *F*_0_. Meanwhile, the fluorescence intensity of compound without single Avelox (*F*_BSA−Avelox_ − *F*_Avelox_) was used as *F* because Avelox has slight fluorescence emission at 343 nm. The fluorescence intensities in this paper were corrected by (1). Otherwise, we recorded the fluorescence spectra when the Δ*λ* value between the excitation and emission wavelengths was stabilized at 15 and 60 nm, respectively.

#### 2.3.2. Probe Displace Experiments

At 293 K, different concentrations of probe drug WF (or CP, DG) were respectively added to the mixture of Avelox-BSA systems. The molar ratio of Avelox and BSA was kept at 1, both of their concentrations are 1.0 × 10^−6^ M in BSA-Avelox systems. The mixtures were diluted to scaled volume with water, mixed thoroughly by shaking, and kept static for 30 min. The excitation wavelength for BSA was 280 nm, with the excitation and emission slit widths set at 5 nm. The fluorescence intensity of BSA only with Avelox was used as *F*_0_ to study the quenching effect of probe drugs on Avelox-BSA system.

## 3. Results and Discussion

### 3.1. Interaction between Avelox and BSA

**Figure 2:**
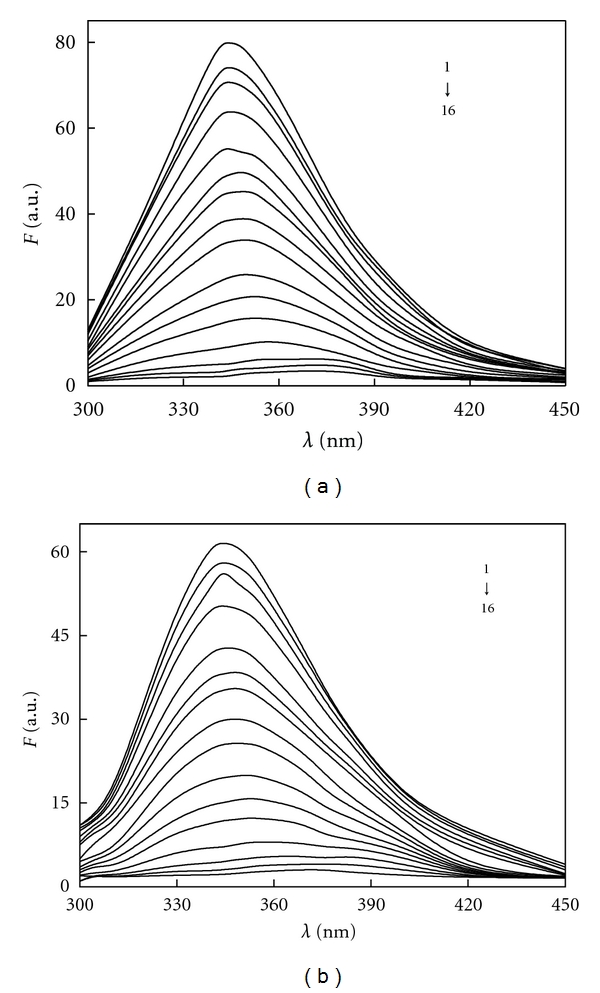
Fluorescence spectra of BSA-Avelox system. *C*_BSA_ = 1.0 × 10^−6^ M, 1~16; *C*_Avelox_ = (0.0, 1.0, 2.0, 4.0, 6.0, 8.0, 10.0, 12.5, 15.0, 20.0, 25.0, 30.0, 40.0, 50.0, 60.0, 70.0) × 10^−6^ M; (a) *λ*_ex_ = 280 nm; (b) *λ*_ex_ = 295 nm;  *T* = 293 K.

**Figure 3:**
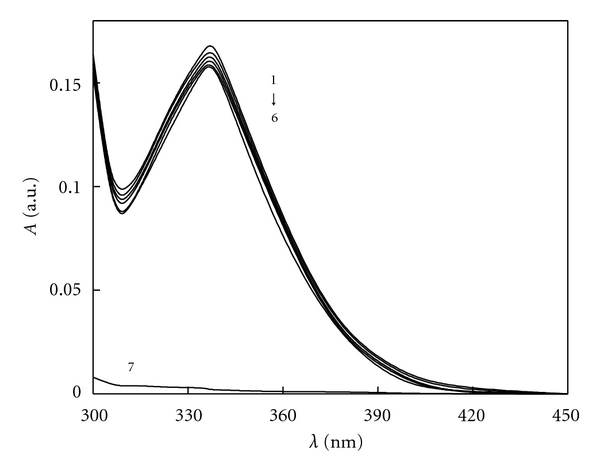
Absorption spectra of Avelox-BSA system From (1) to (6), *C*_Avelox_ = 1.0 × 10^−5^ M, *C*_BSA_ =(0, 0.05, 0.1, 0.5, 0.8, 1.0) × 10^−6^ M, (7): *C*_BSA_ = 1.0 × 10^−6^ M.

Based on Section 2.3.1, we have drawn the fluorescence spectra of BSA as well as different Avelox added into BSA, respectively. When the excitation wavelengths were at 280 nm and 295 nm, the emission peaks for BSA were both located at 343 nm. In this part, we also found that Avelox had fluorescence at 462 nm obviously, but it had weak fluorescence at 343 nm. According to this phenomenon, we scanned the solution on the fluorophotometer with the range 300–450 nm and recorded the fluorescence spectra of BSA-Avelox without Avelox which were shown in Figure 2. As can be seen from Figure 2, the fluorescence intensity of BSA decreased regularly with the addition of Avelox, and the maximum emission wavelength had 9 nm red shifts from 343 nm to 352 nm. This result implied that Avelox could quench the intrinsic fluorescence of BSA strongly, and the interaction between Avelox and BSA resulted from the formation of a ground-state complex between this drug and protein. In addition, the UV absorption intensity of Avelox which located at 336 nm and 308 nm decreased with increased concentrations of BSA (as seen in Figure 3), it could also reveal that the formation of new complex, in addition suggested that the quenching was a static process.

### 3.2. Fluorescence Quenching Mechanism of BSA-Avelox System

Fluorescence quenching can occur by different mechanisms, it may be dynamic quenching, resulting from the collisional encounter between the drug and protein, or static quenching, resulting from the formation of a ground-state complex between the drug and protein. Higher temperature would result in faster diffusion and typically the dissociation of weekly bound complexes, leading to larger amount of dynamic quenching and smaller amounts of static quenching, respectively [5]. In order to confirm the quenching mechanism, the fluorescence quenching data are analyzed by the Stern-Volmer equation [6]:

(2) $$\frac{F_{0}}{F}=1+K_{q}\tau_{0}\left[L\right]=1+K_{\text{sv}}\left[L\right],$$

where *F*_0_ and *F* are the fluorescence intensities in the absence and presence of ligand, respectively. *τ*_0_ is the average lifetime of fluorescence without ligand, which is about 10^−8^ s. *K*_sv_ is the Stern-quenching constant. *K*_*q*_ is the quenching rate constant of biomolecule, and [*L*] is the concentration of the ligand. Based on the linear fit plot of *F*_0_/*F* versus [*L*], the *K*_*q*_ values can be obtained. The calculated results were shown in Table 1. It appeared that the values of *K*_sv_ decreased with the increase in temperature for all systems, which indicated that the probable quenching mechanism of the interaction between BSA and Avelox was initiated by complex formation rather than by dynamic collision [7]. In addition, all the values of *K*_*q*_ were much greater than the maximum scatter collision quenching constant of various quenchers (2 × 10^10^ M^−1^ s^−1^) [8]. This also suggested that the quenching was a static process [9].

For static quenching process, the relationship between the fluorescence intensity and the concentration of quencher can be usually described by deriving (3) [10] to obtain the binding constant (*K*_*a*_) and the number of binging sites (*n*) in most of paper:

(3) $$\log\left(\frac{F_{0}-F}{F}\right)\;=n\log K_{a}+n\log\left\{\left[D_{t}\right]-n\frac{F_{0}-F}{F_{0}}\left[B_{t}\right]\right\},$$

where [*D*_*t*_] and [*B*_*t*_] are the total concentrations of Avelox and protein, respectively. On the assumption that *n* in the bracket is equal to 1, the curve of log⁡(*F*_0_ − *F*)/*F* versus log⁡{[*D*_*t*_]−[*B*_*t*_](*F*_0_ − *F*)/*F*_0_} is drawn and fitted linearly, then the value of *n* can be obtained from the slope of the plot. If the *n* value obtained is not equal to 1, then it is substituted into the bracket and the curve of log⁡(*F*_0_ − *F*)/*F* versus log⁡{[*D*_*t*_] − *n*[*B*_*t*_](*F*_0_ − *F*)/*F*_0_} is drawn again. The above process is repeated again and again till *n* obtained is only a single value or a circulating value. Based on the *n* obtained, the binding constant *K*_*a*_ can be also obtained. In the work, a calculation program was developed. The calculation process can be finished with calculator based on the simple program, and the calculating results can be obtained by inputting *F*, [*D*_*t*_] and [*B*_*t*_]. The calculated results were shown in Table 1.

As seen in Table 1, the values of *n* were approximately equal to 1 at different temperatures, which indicated the existence of just one main binding site in BSA for Avelox. The order of magnitude of binding constants (*K*_*a*_) was 10^4^ indicating the existence of strong interaction between BSA and Avelox. Meanwhile, the values of *K*_*a*_ decreased with the increasing temperature, further suggested that the quenching was a static process [11], hence it led to the reduced of the stability of binary systems. On the other hand, comparing the data of 280 nm with 295 nm, values of *K*_*a*_ and *n* had less difference at different temperatures, it explained that Avelox would mainly quench the fluorescence of tryptophanyl residues from BSA.

### 3.3. Type of Interaction Force of the Binary Systems

Generally, the interaction forces between the small drug molecule and biological macromolecule include hydrogen bond, Van der Waals force, electrostatic interactions, and hydrophobic force [12]. Ross and Subramanian [13] have characterized the sign and magnitude of the thermodynamic parameter, enthalpy change (Δ*H*), free energy (Δ*G*), and entropy change (Δ*S*) of reaction, associated with various individual kinds of interaction. The reaction Δ*H* can be regarded as constant if the temperature changes little. Negative Δ*H* and positive Δ*S* indicate that electrostatic interaction plays a major role in the binding reaction. Positive Δ*H* and Δ*S* are generally considered as the evidence for typical hydrophobic interactions. In addition, Van der Waals force and hydrogen bonding formation in low-dielectric media are characterized by negative Δ*H* and Δ*S* [14]. The thermodynamic parameters can be calculated on the basis of the following equations:

(4) $$R\ln K=\Delta S-\frac{\Delta H}{T},$$

(5) ΔG=ΔH−TΔS.

According to the binding constants *K*_*a*_ of Avelox to BSA at different temperatures above (Table 1), the thermodynamic parameters were obtained conveniently. Therefore, the values of Δ*H*, Δ*S*, and Δ*G* were −1.79 kJ mol^−1^, 85.2 J mol^−1^ K^−1^, and −26.8 kJ mol^−1^ (*T* = 293 K), respectively. The negative value of Δ*G* clarified an automatic reaction between Avelox and BSA. The negative value of Δ*H* and positive value of Δ*S* showed that Avelox mainly bound to BSA by the electrostatic attraction.

### 3.4. Identification of the Binding Sites of Avelox on the BSA

**Figure 4:**
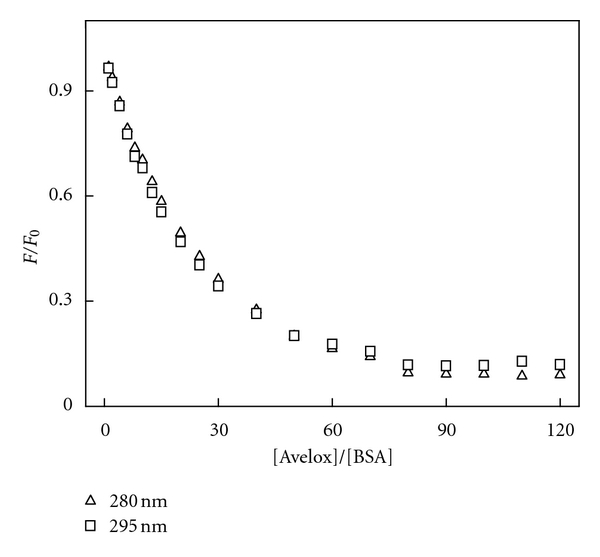
Quenching curves of BSA-Avelox system at *λ*_ex_ = 280 nm and 295 nm *C*_BSA_ = 1.0 × 10^−6^ M, *C*_Avelox_ = 1.0 × 10^−6^~ 1.2 × 10^−4^ M; *T* = 293 K.

 Participation of tyrosine (Tyr) and tryptophan (Trp) groups in drug-serum albumin complexes is assessed using different excitation wavelengths. At 280 nm wavelength, the Trp and Tyr residues in serum albumin are excited, whereas the 295 nm wavelength excites only Trp residues. In the subdomains of BSA, IIA subunit (Trp and Tyr) and IIIA (Tyr) are also considered as the main binding sites for small-drug molecule [15]. Based on the Stern-Volmer equation, comparing the fluorescence quenching of protein excited at 280 nm and 295 nm allows to estimate the participation of Trp and Tyr groups in the complex [16]. As seen in Figure 4, the quenching curves of BSA excited at 280 nm and 295 nm in the presence of Avelox overlap below the molar ratio Avelox : BSA = 80 : 1. This means that if there are fewer than 80 Avelox molecules for 1 BSA molecules, only Trp residues take part in the interaction of Avelox with BSA, and protein fluorescence was quenched by almost 80% at the molar ratio 80 : 1. The quenching of BSA fluorescence exited at 280 nm above this molar ratio was slightly higher than that excited at 295 nm which means that Tyr residues began to take part in the interaction. According to this conclusion, it could be inferred that Avelox molecules only take interaction with Trp residues of BSA at low concentration, whereas both Trp and Tyr residues at high concentration, this conclusion can also expound why the value of *n* is slightly more than 1, which has been shown in Table 1.

BSA has two Trp moieties (Trp-134 and Trp-212) located in subdomains IB and IIA, respectively, and only Trp-212 is located within a hydrophobic binding pocket of the protein which usually bind many small ligands, especially heterocyclic ligands of average size and small aromatic carboxylic acids [17]. In this way, it could confirm that the primary binding site for Avelox was located in subdomain IIA of BSA.

Site marker fluorescent probes are useful for rapid identification of drug binding sites [18, 19]. In order to estimate the drug binding sites on BSA, Sudlow et al. have proposed the percentage of probe replacement method using the following equation [20]:

(6) $$\text{probe displacement}\left(\text{\%}\right)=\frac{F_{2}}{F_{1}}\times 100.$$

**Figure 5:**
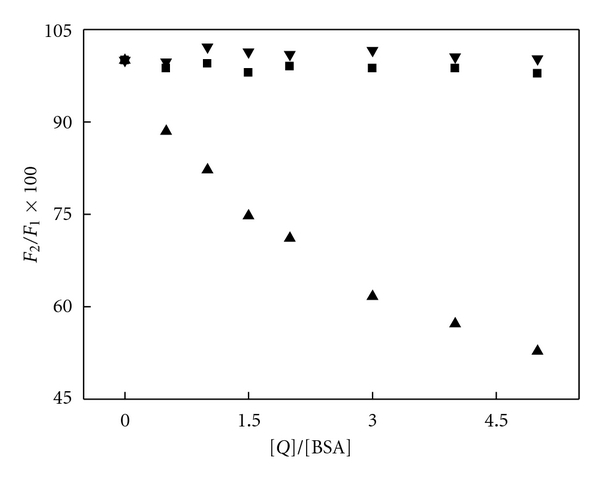
Effect of site maker probe on the fluorescence intensities of BSA-Avelox system *C*_Avelox_ = *C*_BSA_ = 1.0 × 10^−6^ M; *C*_*Q*_ = (0.5, 1.0, 1.5, 2.0, 3.0, 4.0, 5.0) × 10^−6^ M. [*Q*]: ▲ WF; ■ CP; ▾ DG; *λ*_ex_ = 280 nm; *T* = 293 K.

The concentrations of BSA and Avelox were kept constant (1 × 10^−6 ^M), while that different concentrations of various probe drugs each were added into it. In the study of the substitution reaction, *F*_1_ and *F*_2_ are the fluorescence intensities in the absence and presence probe drug. In this paper, we have considered WF, CP, and DG as probe durgs which bind specifically to sites I, II, and III, respectively. The processed results were shown in Figure 5 by (6).

As seen in Figure 5, relative fluorescence intensities decreased obviously with increasing concentration of WF, whereas it almost kept constant with the adding of CP and DG. It indicated that Avelox was pronouncedly displaced by WF rather than CP or DG, hence it could be inferred that the binding site for Avelox was site I which located in sub-domain IIA of BSA.

At room temperature (293 K), control the concentrations of BSA and various probe drugs each at 1 × 10^−6^ M. According to the method described in Section 2.3.1, successively adding different concentrations of Avelox. The experimental data was calculated by (3). The values of binding constants *K*_*a*_ were 4.68 × 10^4^, 5.50 × 10^4^, and 5.37 × 10^4^ M^−1^in presence of WF, CP, and DG, respectively. Compared with the binding constant of Avelox-BSA without any probe drug (5.89 × 10^4^ M^−1^), it can be seen that the binding constant between Avelox and BSA decreased most obviously in the presence of WF, this result indicated that binding of Avelox with BSA was affected by adding WF, which compete the same binding site of BSA. Hence, it also can be proposed that the binding of Avelox primarily occurred in sub-domain IIA (site I).

### 3.5. Hill's Coefficient of BSA-Avelox System

In biochemistry, the binding of a ligand molecule at one site of a macromolecule often influences the affinity for other ligand molecules at additional sites. This is known as cooperative binding. It is classified into positive cooperativity, negative cooperativity, and noncooperativity according to the promotion or inhibition to the affinity for other ligand molecules. Hill's coefficient provides a way to quantify this effect and is calculated graphically on the basis of the following equation [21]:

(7) $$\text{lg}\frac{Y}{1-Y}=\text{lg}K+n_{H}\text{lg}\left[L\right],$$

where *Y* is the fractional binding saturation; *K* is the binding constant and *n*_H_ is the Hill's coefficient. Hill's coefficient is greater than one, which exhibits positive cooperativity. Conversely, Hill's coefficient is less than one, which exhibits negative cooperativity. A coefficient of 1 indicates non-cooperative reaction.

For fluorescence measurement:

(8) $$\frac{Y}{\left(1-Y\right)}=\frac{Q}{\left(Q_{m}-Q\right)},$$

where *Q* = (*F*_0_ − *F*)/*F*_0_; 1/*Q*_*m*_ = intercept of the plot 1/*Q* versus 1/[*L*]. Hill's coefficients were presented in Table 2. The values of *n*_H_ were slightly less than 1 in the systems, both at excitation wavelengths 280 nm and 295 nm at different temperatures, which indicated negative cooperativeness in the interaction of Avelox with BSA, but they were weak. In addition, the values of *n*_H_ were inversely correlated with increasing temperature illustrating the existence of negative cooperativity between Avelox and BSA, that was, the ability of drug bounding to BSA has decreased with the previous ligand (Avelox) bounding to BSA gradually. Furthermore, this negative cooperativity became more powerful with increasing temperature, reducing the amount of Avelox that could be bound to BSA. It was also one of the reasons which led to the reduced *K*_*a*_ with increasing temperature.

### 3.6. Binding Distances between Avelox and BSA

Fluorescence energy transfer is categorized as radiation energy transfer and nonradiation energy transfer. The fluorescence spectrum will deform if radiation energy transfer occurred. The fluorescence spectrum in Figure 2 has no deformation, thus the energy transfer of BSA and Avelox should be nonradiation energy transfer.

**Figure 6:**
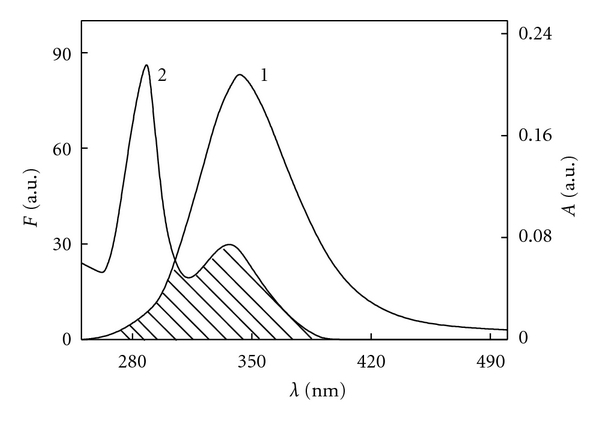
Fluorescence emission spectra for BSA (1) and UV absorbance spectra for Avelox (2). *C*_Avelox_ = *C*_BSA_ = 6.0 × 10^−6^ M; *T* = 293 K.

According to Förster's nonradiative energy transfer theory, energy efficiency *E*, critical energy-transfer distance *R*_0_(*E* = 50%), the energy donor, the energy acceptor distance *r*, the overlap integral, between the fluorescence emission spectrum of the donor, and the absorption spectrum of the acceptor *J* can be calculated by the formulas [22]:

(9) $$E=1-\frac{F}{F_{0}}=\frac{R_{0}^{6}}{\left(R_{0}^{6}+r^{6}\right)},\;$$

(10) $$R_{0}^{6}=8.78\times 10^{-25}K^{2}\Phi N^{-4}J,\;$$

(11) $$J=\frac{\sum F\left(\lambda \right)\varepsilon \left(\lambda \right)\lambda^{4}\Delta \lambda }{\sum F\left(\lambda \right)\Delta \lambda },\;$$

where *K* ^2^ is the orientation factor, Φ is the fluorescence quantum yield of the donor, *N* is a refractive index of the medium, *F*(*λ*) is the fluorescence intensity of the fluorescence donor at wavelength *λ*, and *ε*(*λ*) is the molar absorption coefficient of the acceptor at this wavelength. The overlap of UV-vis absorption spectra of Avelox and the fluorescence emission spectra of BSA (*λ*_ex_ = 280 nm) were shown in Figure 6. Under these experimental conditions, it has been reported that *K* ^2^ = 2/3, *N* = 1.336, and Φ = 0.118 [23]. Thus *J*, *E*, *R*_0_, and *r* were calculated and shown in Table 3. The donor-to-acceptor distance *r* < 7 nm indicated that the energy transfer from BSA to Avelox occured with high possibility [24]. Furthermore, the value of *r* was greater than *R*_0_ in this study which suggested that Avelox could strongly quench the intrinsic fluorescence of BSA by a static quenching mechanism [25]. Moreover, the distance *r* increased, and the energy efficiency *E* decreased with increasing temperature (Table 3), which resulted in the reduced stability of the binary systems and the values of *K*_*a*_.

### 3.7. Conformation Investigation of BSA

**Figure 7:**
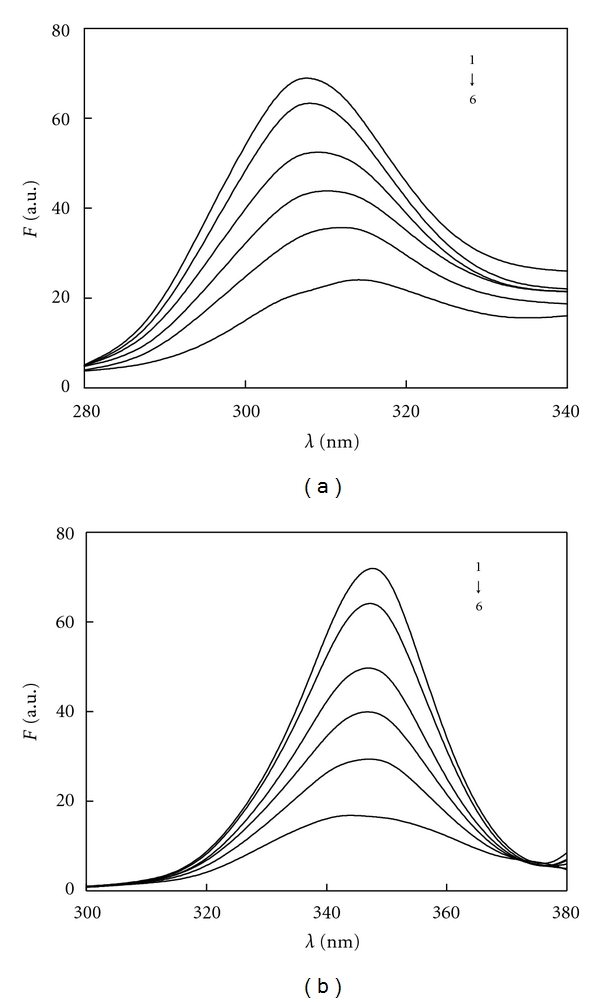
Synchronous fluorescence spectra of BSA-Avelox system *C*_BSA_ = 1.0 × 10^−6^ M, 1 ~ 6 *C*_Avelox_ = (0.0, 2.0, 6.0, 10.0, 15.0, 25.0) × 10^−6^ M; (a) Δ*λ* = 15 nm, (b) Δ*λ* = 60 nm; *T* = 293 K.

Synchronous fluorescence spectra are used to investigate the protein conformational change, as it has been shown to give narrow and simple spectra. For the synchronous fluorescence spectra of protein, when the Δ*λ* value between the excitation and emission wavelengths is stabilized at either 15 or 60 nm, the synchronous fluorescence gives characteristic information for Tyr residues or Trp residues [26]. Because of the red shifts of maximum emission wavelengths of both Tyr and Trp with the less hydrophobic environment, blue shifts of maximun emission wavelengths with the more hydrophobic environment ocurr. These red or blue shifts indicated that the conformation of BSA has been changed [27]. In order to further study the effect on the conformation of BSA, the synchronous fluorescence spectra were measured when the Δ*λ* = 15 nm and Δ*λ* = 60 nm as shown in Figure 7.

It can be seen from Figure 7 that the fluorescence intensities of Tyr and Trp residues decreased regularly with increasing concentration of Avelox. At the same time, the maximum emission wavelength had 4 nm blue shifts at the investigated concentrations range when Δ*λ* = 60 nm, while the maximum emission wavelength had 7 nm red shifts when Δ*λ* = 15 nm. This revealed that the aminoacids microenvironment changed due to the reaction of Avelox and BSA, making the hydrophobicity of Trp residues strengthened, whereas, the hydrophobic environment of Tyr residues more polar. High concentration of drugs makes protein molecules extend, thus reducing energy transfer between aminoacids, reducing the fluorescence intensity [28].

### 3.8. Effect of the Coexistent Drugs on the Binding of Avelox and BSA

According to this method about the percentage of probe replacement method described in Section 3.4, we have taken assays to study the effects of some common antibiotics on the binding of BSA-Avelox system. The concentrations of BSA and Avelox were kept constant (1 × 10^−6^ M), while that different concentrations of various antibiotics each were added into it. In the study of the substitution reaction, *F*_1_ and *F*_2_ are the fluorescence intensities in the absence and presence of antibiotice. It supposed that there was no effect on the binding of BSA-Avelox system when the value of (*F*_2_ − *F*_1_)/*F*_1_ was less than ±5% by (6), that was, this antibiotice could coexist with Avelox. The results showed that it allowed 500 times of gentamicin, thiamphenicol, 300 times of sulfamethoxazole, erythromycin and streptomycin, 100 times of ampicillin, neomycin and kanamycin, 20 times of penicillin G sodium salt and acetyl spiramycin, and 10 times of cefotaxime sodium, cefoperazone sodium, ceftriaxone, and chloramphenicol to coexist with Avelox, but a bit of ciprofloxacin, and norfloxacin could disturb the stability of BSA-Avelox system, making the fluorescence intensity change a lot. It might result that Avelox and ciprofloxacin, norfloxacin all belong to a class of drugs called quinolone antibiotics, having much similar structures, hence, there was certain competition among them for the protein. The competition would lead to binding constant *K*_*a*_ change spontaneously. It could be inferred that there were possible effects on the stayed-time from the blood, as well as plasma concentration and effective concentration, having effects on the efficacy of drugs when the antibiotics such as ciprofloxacin and norfloxacin coexist with Avelox on the binding reaction of BSA. However, aminoglycosides, macrolides, sulfonamides, and penicillin antibiotics have no effects on the binary system, that is, those antibiotics will not affect the transportation of Avelox though BSA when they coexist with Avelox and also have no effects on the efficacy of Avelox.

## 4. Conclusions

In this paper, the interaction of Avelox with BSA was studied at different temperatures by fluorescence spectroscopic methods. The experimental results indicated that Avelox molecular had embedded into the hydrophobic cavity through electrostatic force and was mainly bound to Trp-212 in BSA. The donor-to-acceptor distance *r* was less than 7 nm, indicating nonradiation energy transfer. The negative cooperativity existed in BSA-Avelox system for subsequent ligand. From the synchronous fluorescence spectra, it could be shown that the quaternary conformational change of BSA was induced by the interaction of Avelox with the Trp microregion of BSA molecules. The method-like percentage of probe replacement clearly revealed the existence of effects of common antibiotics on the binding of BSA-Avelox system. The study will extend the use of fluorescence spectroscopy; meanwhile, it provides important insights into the future clinical medicine.

## Figures and Tables

**Table 1:** Quenching reactive parameters of BSA and Avelox at different temperatures.

**λ** **_ex_**	*T*/K	*K*_q_/(M^−1^ s^−1^)	*r*_1_	*K*_a_/(M^−1^)	*n*	*r*_2_
280	293	5.81 × 10^12^	0.9943	5.89 × 10^4^	1.26	0.9971
	303	5.49 × 10^12^	0.9938	5.75 × 10^4^	1.23	0.9903
	313	4.86 × 10^12^	0.9951	5.62 × 10^4^	1.24	0.9913
295	293	6.35 × 10^12^	0.9956	6.02 × 10^4^	1.18	0.9992
	303	6.12 × 10^12^	0.9941	5.89 × 10^4^	1.18	0.9937
	313	5.54 × 10^12^	0.9960	5.75 × 10^4^	1.43	0.9954

*r*_1_ is the linear relative coefficient of *F*_0_/*F* ~ [*L*]; *r*_2_ is the linear relative coefficient of log⁡(*F*_0_ − *F*)/*F* ~ log⁡{[*D*_*t*_] − *n*[*B*_*t*_](*F*_0_ − *F*)/*F*_0_}.

*K*_*q*_ is the quenching rate constant; *K*_*a*_ is the binding constant; *n* is the number of binding site.

**Table 2:** Hill's coefficients *n*_H_ of BSA-Avelox at different temperatures.

*T*/K	*λ*_ex_/280 nm		*λ*_ex_/295 nm	
	*n_H_*	*r*_3_	*n_H_*	*r*_3_
293	0.89	0.9938	0.86	0.9932
303	0.74	0.9959	0.72	0.9953
313	0.70	0.9913	0.69	0.9906

*r*_3_ is the linear relative coefficient of lg[*Y*/(1–*Y*)] ~ lg[*L*].

**Table 3:** Parameters of *E*, *J*, *r*, and *R*_0_ between Avelox and BSA at different temperatures.

* T*/K	*E *(%)	*J*/(M^−1^ cm^3^)	*R*_0_/nm	*r*/nm
293	28.04	1.07×10^−14^	2.48	2.90
303	26.66	1.04×10^−14^	2.47	2.92
313	25.60	1.02×10^−14^	2.46	2.95

*R*_0_ is the critical distance when *E* is 50%; *r* is the distance between acceptor and donor; *J* is the overlap integral between the fluorescence emission spectrum of donor and the absorption spectrum of the acceptor.
